# Navigation-Guided Endoscopy Combined with Deep Lateral Orbitotomy for Removal of Small Tumors at the Lateral Orbital Apex

**DOI:** 10.1155/2018/2827491

**Published:** 2018-11-25

**Authors:** GuangMing Zhou, Xin Ju, Bo Yu, YunHai Tu, JieLiang Shi, EnDe Wu, WenCan Wu

**Affiliations:** Minimally Invasive Surgical Center, The Eye Hospital of Wenzhou Medical University, Wenzhou, Zhejiang, China

## Abstract

**Purpose:**

To evaluate the efficacy, feasibility, and safety of the navigation-guided endoscopy combined with deep lateral orbitotomy for removal of small tumors at the lateral orbital apex.

**Design:**

A retrospective, noncomparative case series.

**Methods:**

Retrospective analysis of ten patients (10 eyes) with small tumors at the lateral orbital apex comprised navigation-guided endoscopy combined with deep lateral orbitotomy at the Eye Hospital of Wenzhou Medical University from November 2015 to November 2017. In each case, the indication of surgery was existing or imminent visual impairment due to the tumor. The removal was believed to be complete if the mass was removed intactly during the surgery. The tumor character was confirmed by pathological examination after surgery. Patients were followed up to 3 months after surgery. Best corrected visual acuity before and after surgery was compared.

**Results:**

All tumors were completely removed by the navigation-guided endoscopic approach. The mean preoperative best corrected visual acuity was 6/15 (95% confidence interval (95% CI) 6/40–6/8.5), and the mean postoperative best corrected visual acuity was 6/10 (95% CI 6/15–6/7.5). 5 of 7 (71%) patients with vision loss gained visual improvement in different degrees after surgery, and the rest of the patients had preoperative best corrected visual acuity. Visual field of all patients also improved. 8 cavernous hemangiomas and 2 schwannomas were confirmed postoperatively by pathology. 4 patients accompanied with limitation of eye abduction, which recovered spontaneously in an average of 4 weeks. No other serious complications occurred.

**Conclusions:**

Navigation-guided endoscopy combined with deep lateral orbitotomy seems to be a feasible, efficient, and safe approach for removing small tumors at the lateral orbital apex. This trial is registered with ChiCTR1800019244.

## 1. Introduction

The orbital apex is an important compartment containing very delicate structures essential for vision. Tumors located at this place were often undetected for a long period and may lead to compression of the optic nerve and loss of vision. Thus, operation should be performed at the very early stage of the disease. However, the compact space of the orbital apex increases the difficulties of the surgical approach.

In order to keep the integrity of the anatomical structures and functions of the orbit, proper surgical exposure needs to be achieved during the surgery. Several approaches have been used in removal of the apical tumor by ophthalmologists and neurosurgeons for decades. But the choice of operation methods for removing small tumors located at the orbital apex remains unsure by most ophthalmologists because of the poor symptom improvement and inevitable iatrogenic injury [1–4]. Recently, an endoscopic transnasal approach for the apical tumor has been reported in some literatures [5–9] which has gained great outcome. However, this approach was made for well-defined tumors located medial or inferomedial to the orbital apex, especially close to the medial orbital wall. It was helpless for tumors located at the lateral orbital apex. Furthermore, tumors, especially small tumors located at the lateral orbital apex, are quite hard to be explored and identified only by endoscopy. The small tumor at the lateral orbital apex was still, so to speak, a “nightmare” for surgeons.

In this study, we developed an endoscope combined with the navigation to facilitate the deep lateral orbitotomy for removing small tumors at the lateral orbital apex. We report 10 cases of small tumors, including 8 cavernous hemangiomas and 2 schwannomas, at the lateral orbital apex which were treated via this approach. Obvious visual improvement was gained after surgery.

## 2. Methods

### 2.1. Clinical Data

From November 1, 2015, to November 1, 2017, altogether 10 cases with small tumors located at the lateral orbital apex were identified from the electronic medical record system of the Eye Hospital of Wenzhou Medical University. All cases were diagnosed by high-resolution computed tomography (HRCT) or magnetic resonance imaging (MRI). Best corrected visual acuity (BCVA), visual field, pupil reflex, eye movement, and complete status of the anatomy of both eyes were checked preoperatively and postoperatively in all cases. Cases with visual decline, imminent visual impairment, or visual field defect caused by the tumor were referred to surgery. Patients were followed up to 3 months after surgery. Informed consent was obtained from all patients before surgery.

### 2.2. Surgery Procedures

**Figure 1:**
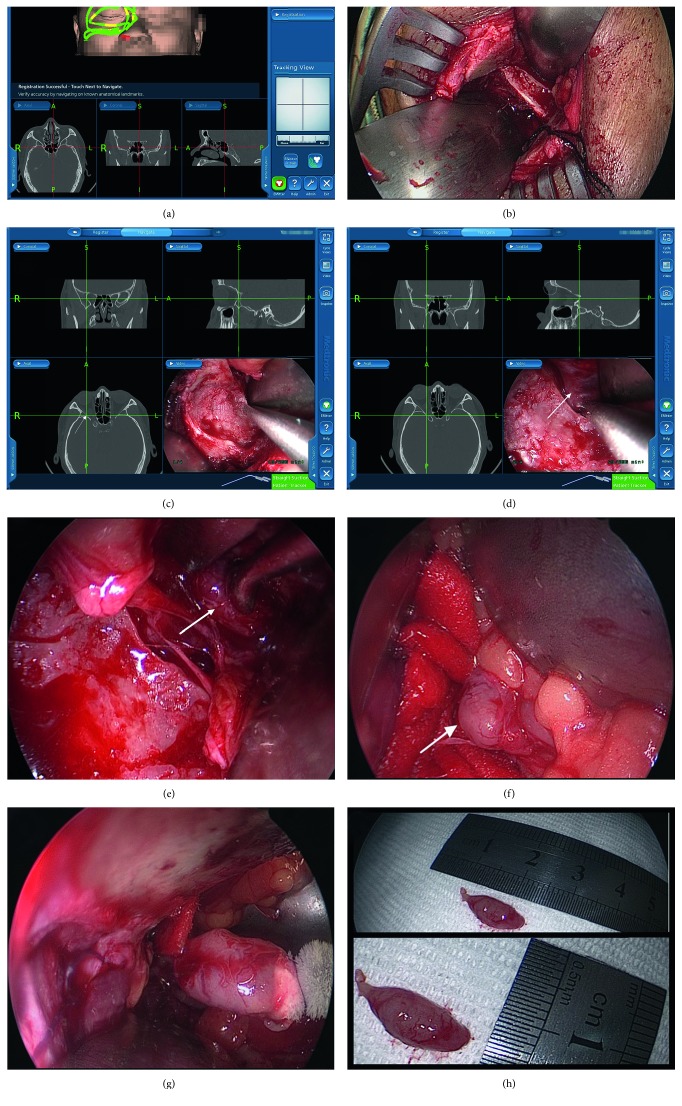
Surgical view of removal of the tumor at the lateral orbital apex with a navigation-guided endoscope. (a) Matching the patient's anatomical landmark with imaging date. (b) Lateral orbitotomy removed the lateral wall. (c, d) Removing the lateral wall to expose the apex and access the tumor (white arrow) under a navigation-guided endoscope. (e, f) Intraconal tumor was identified and exposed fully (white arrow). (g) The tumor was removed integrally by cryoextraction. (h) Intact tumor size: 13 ∗ 8 mm.

All procedures were performed under general anesthesia. The surgeon matched the anatomical landmark of the patient with the preoperative imaging data via the navigation probe and then selected suitable skin incision depending on the location of tumors. For the lateral orbital tumor, we approached the tumor from a horizontal skin incision of the lateral canthus; for the superolateral tumor, we chose S-shaped skin incision, which included inferior margin of the eyebrow and lateral canthus; for the inferolateral tumor, we approached the tumor from a skin incision of the lateral canthus which extended to the lower eyelid margin. After the skin incision, a superior and inferior dissection was made to reach supraorbital and infraorbital margins, respectively, to expose the lateral wall; an electro-saw was used to cut the zygoma, and then it was fractured laterally with the use of a rongeur. For the further adequate exposure of the orbital apex, a navigation-guided microdrill and rongeur were used to remove the greater wing of the sphenoid bone posteriorly until the dura mater was encountered; the extent of the bone removal was dependent on the location and size of the tumor and the expected exposure required; the osteotomized bone was preserved by the saline gauze. After this, intraorbital dissection was performed with a standard endoscope (diameter 4.0 mm, 45 degrees) under direct vision, which provides a high resolution, illumination, and magnification, to explore and expose the lesion precisely; the endoscopic approach was also guided by navigation. After the tumor was visualized, the surgeon grasped the tumor by using a long forceps and then separated the tumor from the orbital apex by gradual blunt and gentle dissection from posterior to anterior. Any excessive traction and grasp were forbidden, especially during the dissection of schwannoma; our experience indicates that, due to the weak capsule of schwannoma, any violent operation may cause schwannoma rupture which may lead to tumor recurrence, so cryoprobe could be considered to ensure the integrity of tumors. Moreover, a long-standing tumor at the apex always has adherence to the surrounding tissue; any reckless manipulation may lead to irreversible consequences. If there was strong adherence to the adjacent tissue, in order to ensure to remove tumors as thoroughly as possible, piecemeal resection could be made for cavernous hemangioma and intracapsular removal could be made for schwannoma; besides, subtotal excision, radiotherapy, or apical decompression also should be considered strongly when faced with an extremely adherent lesion during surgery. To avoid any iatrogenic injury, all above manipulation needs to be done with the use of an endoscope, and the surgeon should dissect the orbit prudently and mildly by blunt dissection under direct vision, while the assistant pulled the lateral rectus and protected orbital contents. Moreover, navigational positioning must be performed repeatedly before apical manipulation. During the whole procedure, the pupil of the operated eye was closely observed. If the mydriasis appeared during this procedure, which indicated possible optic nerve injury by mechanical damage or vascular compromise, all traction was released immediately and more precise dissection must be relocated. There was a clear demarcation of the tumor that separates from the adjacent tissue, so the mass was removed integrally. After tumor resection, the surgeon reinspected the orbit seriously to ensure no tumor remains, and then the whole surgical field was stanched thoroughly and carefully by the biofluid hemostatic membrane and bipolar cautery. Oxydol and tobramycin dexamethasone brine douched the surgical cavity in order. The final step was to reconstruct the orbital structure; the surgeon restored the orbit as possible as original to ensure normal function, and the osteotomized bone was replaced in the prepositioned area by using a titanium screw and sheet. The subcutaneous tissue and skin were closed with suture. Pressure bandaging followed tobramycin dexamethasone eye ointment covering incision. Resected tumors further confirmed the pathological character (Figure 1).

Postoperative management included intravenous methylprednisolone (500 mg) for 3 days, intravenous broad-spectrum antibiotics for 5 days, and an additional neurotrophic drug. The patients were positioned to lie in bed for 4–7 days after surgery, in a 30-degree tilt position with head up and feet down. Any intense activity, collision, and squeeze were prohibited for the first 6 months after surgery. Vision, proptosis, and eye movement were supervised closely. Because all the tumors were well defined and were removed in toto during the surgery, the removal was believed to be complete and no further imaging examination was conducted.

## 3. Results

**Figure 2:**
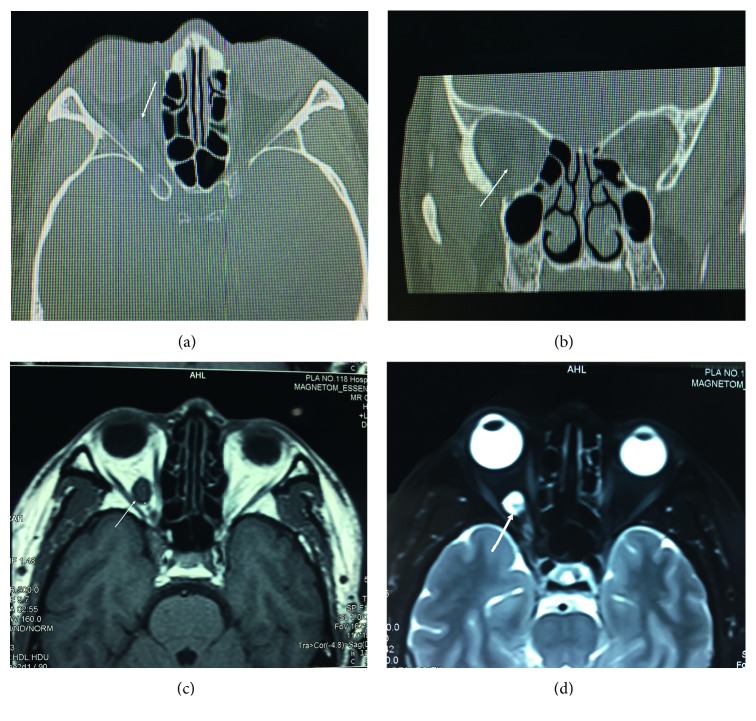
Computed tomography (CT) and magnetic resonance imaging (MRI) of the patient with schwannoma at the apex. (a, b) Orbital CT images showed a suborbicular and well-defined mass with isodensity mass located at the right inferolateral apex. (c) MRI T1-weighted images showed an isointensity mass at the right apex. (d) MRI T2-weighted images showed a high-intensity mass (white arrows).

Ten cases were included in our study. Among these 10 patients, 2 were male and 8 were female. The age ranged from 24 to 70, with a mean age of 44.3 ± 17.9 years. 7 patients had insidious onset and complained of slowly progressive visual decline. 3 patients (patients 5, 6, and 9) without visual decline were found to have tumor by medical examination with an average onset of 17.2 ± 17.7 months (range 1–60 months) before presentation. All patients showed visual field defect before surgery. 5 patients had dilated pupil with a relative afferent pupillary dysfunction (RAPD). 4 patients' optic disc was pale with a clear margin and 5 patients were without any abnormality of the pupil and optic disc. No patients had other common symptoms related to orbital tumor, such as significant protopsis, diplopia, or limitation of ocular movement. In imageology, the cavernous hemangiomas and schwannomas showed similar features as a well-defined mass with isodensity on HRCT. MRI showed slightly low density or isointensity on T1WI and high intensity on T2WI. Fat suppression and tissue enhancement technology can differentiate the type of tumor further. Accurate clinical diagnosis can be confirmed by all these clinical features; however, pathology was still the most reliable (Figure 2). All tumors were removed completely. The size of tumors ranged from 10 ∗ 10 mm to 18 ∗ 18 mm, including 8 cavernous hemangiomas and 2 schwannomas in pathology. The mean operation time was 1.96 ± 0.61 hours.

Five of 7 (71%) patients with vision loss gained BCVA improvement after surgery. The mean BCVA before and after operation was 6/15 (95% CI 6/40–6/8.5; range 6/300–6/6) and 6/10 (95% CI 6/15–6/7.5; range 6/120–6/6), respectively. The rest of the patients did not have improvement in VA after surgery. The visual field showed different degrees of recovery in each patient, while the appearance of the optic disc and positive RAPD remained the same (Table 1). The most common complication was abduction limitation in 4 patients because of paresis of the lateral rectus. All of them recovered spontaneously in an average of 4 weeks after surgery. No patient complained of the change in periorbital appearance. No other serious complications occurred.

## 4. Discussion

To the best of our knowledge, the small tumor at the orbital apex was not exceedingly rare. It was extremely harmful to visual function due to optic nerve compression. In our study, most of the cases (7/10) complained of impaired vision which was insidious in onset and deteriorated afterward. A careful investigation of the medical history revealed that most patients had temporary blurred vision at the very beginning but did not pay enough attention to it until the vision deteriorated. Part of the patients were found to have tumor by medical examination always accompanied with visual field defect which was not noticed. Those patients showed positive RAPD and pale optic disc, indicating compressive optic neuropathy. Although cavernous hemangiomas and schwannomas are benign and slowly progressive, they have the tendency to result in irreversible vision loss. Therefore, early intervention for small tumors at the apex should be taken before visual impairment.

Surgical access to intraorbital lesions was demanding. The maintenance of the orbital structure and maintenance of function were the major points of interest. Selection of a proper approach to intraorbital tumor relies on multiple factors, such as the location, size of the lesion, and the probable pathology anticipated [10]. However, a narrow and complex orbital apex restricted observation and manipulation; our experience indicates that the key to successful operation is adequate exposure of the operative field, which provides us with possibility to make exact identification of vital structures and possible variation. Ophthalmologists generally chose transorbital approaches for resecting orbital tumors, but this approach was limited by poor visualization and always accompanied with little visual improvement and major injury. Thus, many surgeons recommend neurosurgical approaches for removing apical tumors; the transcranial approach was the preferred choice for most neurosurgeons. Although the transcranial approach provides an impressive surgical exposure, the added operative time, injury, and brain manipulation were inevitable. In recent years, we have commonly applied endoscopic transnasal surgery for small apical tumors [5, 9]. With the help of superiority of the transnasal endoscope, we removed the tumor completely and safely with delicate manipulation. Despite these advances in the surgical technique, this approach was merely designed for the tumor which was located medially or inferomedially to the orbit, and removal of the small tumor at the lateral orbital apex remains challenge. In addition, to our experience, the location and adjacent relationship of apical tumors was not always identified exactly on preoperative imaging; only endoscopy is not enough for the apical small tumor, since the tumor is too deep and small to be identified. Considering the above difficulties, we performed the deep lateral orbitotomy combined with a navigation-guided endoscopy to remove the lateral apical tumor.

The lateral orbitotomy was a widely used approach in orbital surgery and especially suitable for orbital apical tumors [11, 12]. This approach required a minor skin incision, which can be largely hidden within the natural skin crease, producing less wound and excellent aesthetic results. However, the surgical exposure obtained from a sole lateral orbitotomy has limited its clinical adaptability for apical tumors. Thus, we propose that the surgical exposure can be significantly enhanced by instruments. Endoscopic visualization provided surgeons with the possibility to reach the orbital apex directly without brain manipulation and with minimal neurovascular disturbance. Moreover, endoscopes were more advantageous, serving minimal eyeball retraction. With the endoscopic help, we can minimally manipulate in an extremely narrow space under direct vision. Less manipulation ensures less injury. Navigation systems use special computer software, the image data of patients before surgery, and three-dimensional (3D) reconstruction through electromagnetic induction position on the operation instruments for accurate location. With the guidance of 3D images, the surgeon can determine the exact position and extent of the lesion. Navigation can improve surgical accuracy and safety and reduce serious complications. Navigation-guided surgery has been applied commonly in otolaryngology, neurosurgery, orthopedics, and orbital disease [13–18] and gained remarkable effect. However, we are unaware of previous reports of a navigation-guided endoscopy combined with the deep lateral orbitotomy approach as described here and could find few references on the PubMed. Our study shows that this approach takes full advantage of navigation and endoscope, maybe a promising alternative approach in the management of small tumors at the lateral orbital apex.

In our opinion, the central concept of operation is the dissection technique of tumors. The surgeon must be flexible in having the ability to assess the tumor location and character and then select the superior resection technique. There were some similarities between cavernous hemangiomas and schwannomas [19, 20], such as being well defined, fully encapsulated, and loosely adherent. These characteristics determine that the tumor is easy to remove completely, except some apical tumors. But the dissection technique for these two tumors was not identical. Cavernous hemangiomas with a tough capsule can usually be removed by grasp and traction. However, in our experience, the capsule of schwannoma was exceedingly fragile, even the “finger dissection” may break it occasionally, and tumor rupture may lead to tumor recurrence, so immoderate grasp and traction is undoubtedly forbidden. Hence, we proposed that cryoprobe could be considered for integrity of tumors. Besides, orbital schwannomas are tumors originating from the intraorbital nerve except optic nerve [20]; excessive traction may lead to serious damage. The apical tumor often adheres to adjacent; the adherence of the tumor at the apex was a pivotal factor in determining whether the safe and efficient dissection could be performed. Multiple delicate dissections should be taken under endoscope to prevent irretrievable outcome from improper operation, and alternate conservative treatments, such as radiotherapy in the form of gamma knife, also have been reported [21]. Of particular note is that the pupil of the operated eye should be closely monitored during the procedure; it is a significant signal of optic nerve injury. Additional attention should be paid to prevent vascular damage, and unexpected damage of major blood vessels may cause uncontrollable bleeding. In our study, we found that we can avoid heavy bleeding by subtle manipulation and control any bleeding by gauze packing with or without dilute adrenaline (<1 : 100000), bipolar cautery, or biofluid hemostatic membrane. It should be noted that cautery should be avoided at the apex because the thermal damage may compromise the vascular supply to the optic nerve or injure the nerve directly. We were also cautious with adrenaline, which may lead to ischemia, even though it was diluent. Fortunately, owing to the advantage of this approach and our precaution and experience, we have never underwent any terrible accident.

All tumors in our study appeared to be completely removed. All cases in our study gained visual improvement or visual preservation after surgery. We found that the visual improvement was associated with the duration of symptoms. Patients with a shorter duration of symptoms had more vision recovery after surgery (Table 1). In tumor cases, the optic nerve was compressed gradually by the growing lesion. Thus, short duration of symptoms always means short-time optic nerve compression, whose function may be recovered after removing the lesion. Furthermore, a long-standing apical tumor may become more adherent to the apical structure, increasing dissected difficulty and risk. It was noteworthy that 2 patients (patients 4 and 10) with the tumor adherent to the apex firmly in longer duration gained no visual improvement, but 1 patient (patient 3) accompanied with mild compression of the optic nerve in longest duration gained greatest visual recovery and fewest visual field improvement after surgery. So we believed that the location of the tumor in relation to the orbital apex may be a more important indication of the severity of compressive optic neuropathy, which can be used as a prognostic factor to assess the vision recovery. We also evaluated the relation between the preoperative BCVA and the tumor size but did not find any correlation.

As regards surgical complications, the navigation-guided endoscopic surgery also showed conspicuous advantage. The orbital tumor removed by the traditional approach is frequently accompanied with serious complications, such as heavy bleeding, cerebrospinal leak, or visual loss, which were thought to be caused by mechanical trauma to the vessel, rectus, or nerve during surgery [22–25]. Because the apical tumor is even more difficult to access, we deduced that the complication associated with the traditional approach is more likely to occur. Fortunately, none of the serious complication occurred in our study using a navigation-guided endoscopic approach, and only 4 patients experienced temporary limitation of eye abduction but recovered spontaneously in an average of 4 weeks; this may be because of paresis of the lateral rectus caused by intraoperative traction. Thus, we believed navigation-guided endoscopic surgery was a safe approach.

However, we found that there are still some problems of the clinic application of the navigation. On the one hand, navigation was based on the preoperative image examination entirely, cannot show intraoperative changes completely, and inevitably had some error [26]. Thus, the surgeon needs to be familiar with the tumor at the apex. All manipulation should be under the direct vision rather than depending on the instrument completely. On the other hand, headset loosing may affect the location of the structure. Regular check of the headset is quite necessary. Rematching should be done when overlarge deviation was detected. Moreover, the navigation increased the surgical cost. Thus, for the purpose of better clinical results, the surgeon must select most suitable treatment options after comprehensive evaluation.

## 5. Conclusions

Overall, we believed that navigation-guided endoscopic surgery is a promising method for removal of small tumors at the lateral orbital apex, which can increase surgical efficiency and decrease surgical risk. However, the surgical procedure requires sufficient caution, technique, and experience and should be performed only after comprehensive assessment. This study only provides preliminary results. Limitations of our study were inevitable due to its retrospective nature. We failed to record color vision tests and VEP in our cases. Large-sampled controlled prospective research studies are needed to further prove rationality of navigation-guided endoscopic surgery for cases with small tumors at the lateral orbital apex and to work out the exact surgical indication.

## Figures and Tables

**Table 1:** Clinical date of 10 patients with tumors at the lateral orbital apex.

Number	Sex	Age (years)	History (months)	BCVA		RAPD		Optic disc		Visual field		Tumor size (mm)	Tumor type
				Pre	Post	Pre	Post	Pre	Post	Pre	Post		
01	F	26	6	6/300	6/7.5	+	+	Pale	Pale	Irregular defect	Improved	10 ∗ 12	Cavernous hemangioma
02	F	24	12	6/300	6/120	+	+	Pale	Pale	Irregular defect	Improved	10 ∗ 15	Cavernous hemangioma
03	F	61	60	6/300	6/7.5	+	+	Normal	Normal	Irregular defect	Improved	20 ∗ 15	Cavernous hemangioma
04	F	70	24	6/30	6/30	−	−	Normal	Normal	Irregular defect	Improved	18 ∗ 18	Cavernous hemangioma
05	F	68	3	6/6	6/6	−	−	Normal	Normal	Irregular defect	Improved	20 ∗ 10	Cavernous hemangioma
06	M	42	6	6/7.5	6/7.5	−	−	Normal	Normal	Irregular defect	Improved	10 ∗ 10	Cavernous hemangioma
07	F	32	12	6/10	6/6	+	+	Pale	Pale	Irregular defect	Improved	10 ∗ 15	Cavernous hemangioma
08	F	50	1	6/12	6/10	+	+	Pale	Pale	Irregular defect	Improved	12 ∗ 10	Cavernous hemangioma
09	M	47	24	6/7.5	6/7.5	−	−	Normal	Normal	Irregular defect	Improved	13 ∗ 8	Schwannoma
10	F	23	24	6/40	6/40	−	−	Normal	Normal	Irregular defect	Improved	20 ∗ 15	Schwannoma

BCVA: best corrected visual acuity; pre: preoperative; post: postoperative; RAPD: relative afferent pupillary dysfunction.

## Data Availability

The data used to support the findings of this study are included within the article.
